# Improving the Yield and Quality of Daptomycin in *Streptomyces roseosporus* by Multilevel Metabolic Engineering

**DOI:** 10.3389/fmicb.2022.872397

**Published:** 2022-04-18

**Authors:** Zhong-Yuan Lyu, Qing-Ting Bu, Jiao-Le Fang, Chen-Yang Zhu, Wei-Feng Xu, Lie Ma, Wen-Li Gao, Xin-Ai Chen, Yong-Quan Li

**Affiliations:** ^1^First Affiliated Hospital and Institute of Pharmaceutical Biotechnology, Zhejiang University School of Medicine, Hangzhou, China; ^2^Zhejiang Provincial Key Laboratory for Microbial Biochemistry and Metabolic Engineering, Hangzhou, China

**Keywords:** *Streptomyces roseosporus*, daptomycin, titer, quality, multi-level metabolic engineering

## Abstract

Daptomycin is a cyclic lipopeptide antibiotic with a significant antibacterial action against antibiotic-resistant Gram-positive bacteria. Despite numerous attempts to enhance daptomycin yield throughout the years, the production remains unsatisfactory. This study reports the application of multilevel metabolic engineering strategies in *Streptomyces roseosporus* to reconstruct high-quality daptomycin overproducing strain L2797-VHb, including precursor engineering (i.e., refactoring kynurenine pathway), regulatory pathway reconstruction (i.e., knocking out negative regulatory genes *arpA* and *phaR*), byproduct engineering (i.e., removing pigment), multicopy biosynthetic gene cluster (BGC), and fermentation process engineering (i.e., enhancing O_2_ supply). The daptomycin titer of L2797-VHb arrived at 113 mg/l with 565% higher comparing the starting strain L2790 (17 mg/l) in shake flasks and was further increased to 786 mg/l in 15 L fermenter. This multilevel metabolic engineering method not only effectively increases daptomycin production, but can also be applied to enhance antibiotic production in other industrial strains.

## Introduction

Daptomycin is a cyclic lipopeptide antibiotic produced by *Streptomyces roseosporus* (*S. roseosporus*) *via* non-ribosomal peptide synthetases (NRPSs; Debono et al., 1988). It is the best substituent to vancomycin, due to its high antibacterial activity to Gram-positive pathogens, including vancomycin-resistant *Staphylococcus aureus* (*S. aureus*), methicillin-resistant *S. aureus*, and vancomycin-resistant *Enterococci* (Akins and Rybak, 2001). Therefore, how to increase daptomycin production has attracted more attention of researchers.

Daptomycin biosynthetic gene cluster (BGC) belongs to the classical NRPS pathway. Daptomycin synthesis begins with the activation of decanoic acid by DptE, which then transfers the acid onto DptF. The condensation reaction between acid and tryptophan is catalyzed by DptA. Next, under the action of DptA, DptBC, and DptD, the remaining 12 amino acids are condensed and connected to the being synthesized peptide chain in turn. Finally, under the catalysis of the thioesterase domain contained in DptD, through the condensation of Thr4 and Kyn13, the peptide chain is cyclized to produce daptomycin (Supplementary Figure 1; Miao et al., 2005; Wittmann et al., 2008). Kynurenine (Kyn) is one of the main non-proteinogenic amino acid precursors in daptomycin biosynthesis, which is an intermediate produced in the tryptophan degradation pathway. In *S. roseosporus*, tryptophan degrades to generate Kyn, catalyzed by tryptophan-2,3-dioxygenase (DptJ and TDO) and N-formyl kynurenine formamidase, successively. Then, Kyn is converted to anthranilate by kynureninase encoded by the *kyn* gene (Liao et al., 2013) or *orf3244* (predicted by us in this study). According to our prediction in this study, kynurenine is also a substrate for the catalysis of the protein encoded by gene *orf3242*. Overexpression of *dptJ* and disruption of *kyn* made the production of daptomycin an increase of 110 and 30%, respectively (Liao et al., 2013). Decanoic acid (DA) is another important precursor in the production of daptomycin (Wittmann et al., 2008). Daptomycin yield was increased by 40% compared with the wild-type through developing a DA-resistant (DAR) *S. roseosporus via* a sequential adaptation method (Lee et al., 2016). Oxygen is also a pivotal factor in the synthesis of secondary metabolites by *Streptomyces*. Sufficient oxygen helps in better growth and differentiation of mycelium, thereby facilitating product accumulation. Increasing stirring speed or airflow was usually used to improve the dissolved oxygen in the culture broth (Wentzel et al., 2012). However, because of filamentous nature of *Streptomyces*, higher agitation speed may damage mycelium itself and then affect the production of secondary metabolites (Ferraiuolo et al., 2021). Vitreous hemoglobin (VHb) is an oxygen-binding protein that helps microorganisms to grow under low dissolved oxygen conditions. Its heterologous expression successfully increased metabolite productivity by enhancing oxygen utilization efficiency (Horng et al., 2010; Zhu et al., 2011, Li et al., 2016; Mironczuk et al., 2019). This is a great alternative to boost the oxygen supply to prevent hyphae damage.

Furthermore, daptomycin biosynthesis is under the control of several regulators. Three cluster-situated regulators, namely, DptR1, DptR2, and DptR3, are all required for daptomycin production (Wang et al., 2014; Zhang et al., 2015, Yu et al., 2020). Additionally, other factors far away from the daptomycin BGC also play a regulatory role. arpA, a homolog of the A-factor receptor, negatively controls morphological development and daptomycin production in *S. roseosporus*. Removal of *arpA* can increase daptomycin production (Mao et al., 2015). In 2018, a new transcriptional regulator phaR was identified in *S. roseosporus*, which negatively regulates the expression of daptomycin BGC. Deletion of *phaR* led to an approximately 43% increase of daptomycin production in fed-batch fermentation (Luo et al., 2018b). Moreover, the daptomycin BGC has been captured and used to improve the production of daptomycin in its native or heterologous hosts. For instance, daptomycin BGC was cloned from *S. roseosporus* NRRL 15998 and introduced to the strain itself, leading to an increase of daptomycin yield (Du et al., 2015). In addition, a 65-kb daptomycin BGC was expressed heterologously in *Streptomyces coelicolor* (*S. coelicolor*) M511 and the production of daptomycin and its derivatives reached 28.9 mg/l (Choi et al., 2019). Besides, it will also be accompanied by the output of byproducts in the secondary metabolic process, which will affect the separation and purification process, resulting in an increase in industrial costs (Wang et al., 2019). Notably, pigments are sometimes found as byproducts of the antibiotic biosynthesis process. Reseachers improved the fidaxomicin production by removing the orange pigment byproduct in an industrial strain *Actinoplanes deccanensis* YP-1 (Li et al., 2021c).

**FIGURE 1:**
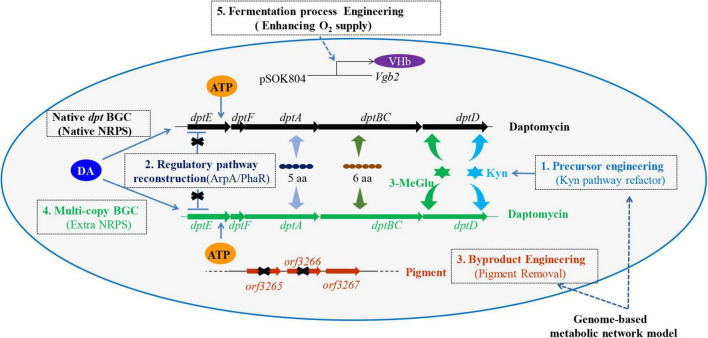
The overview map of metabolic engineering strategies for improving daptomycin production and quality in *Streptomyces roseosporus* (*S. roseosporus*) L2790. In this study, the multilevel engineering strategies included enhancement of Kyn precursor supply (level 1), reconstruction of the regulatory pathway (level 2), byproduct engineering (level 3), multicopy of BGC (level 4), and fermentation process engineering (level 5). Kyn, kynurenine; 3-MeGlu, 3′-methylglutamic acid; 6 aa, six amino acids (Orn 6, Asp 7, D-Ala 8, Asp 9, Gly 10, D-Ser 11); 5 aa, five amino acids (Trp 1, D-Asn 2, Asp 3, Thr 4, Gly 5); DA, decanoic acid; NRPS, non-ribosomal peptide synthetase; BGC, biosynthetic gene cluster; VHb, *Vitreoscilla* hemoglobin; *Vgb2*, a gene encoding protein VHb; pSOK804, an overexpression vector; *dptE*, *dptF*, *dptA*, *dptBC*, and *dptD* are the genes locating in daptomycin BGC and being involved in the biosynthesis of daptomycin; for level 2, the “X” indicates a block of negative regulation; for level 3, the “X” indicates deletion of the relevant genes.

Although many strategies have been applied to enhance the production of daptomycin, most of them are only confined to the manipulation of single factor, such as regulation or precursor supply. Actually, the biosynthesis of daptomycin is influenced by multiple (external and internal) factors. Therefore, to achieve a higher yield of daptomycin, various favorable factors need to be considered and integrated. Here, a combinatorial metabolic engineering measure was implemented to construct daptomycin-overproducing strains (Figure 1). To this end, the supply of precursor Kyn was enhanced by deletion of *orf3242* and *orf3244* (level 1). Next, pleiotropic regulator genes, including *arpA* and *phaR*, were knocked out to release the negative regulation (level 2). Then, partial knockout of genes participating in pigment biosynthesis blocked the pigment production (level 3) and this is the first time to remove red pigment for daptomycin production. Subsequently, extra copy of daptomycin BGC was integrated into L2796 (level 4). Finally, heterologous VHb was expressed in L2797 to alleviate the low dissolved oxygen limitation during large-scale fermentation with daptomycin production up to 786 mg/l (level 5). Such a multilevel design and integration may be offering a method to improve the quality and yield of products in many industrial bacteria.

## Materials and Methods

### Strains and Media

*Streptomyces roseosporus* L2790 was a daptomycin-producing strain, which was stored in our laboratory. *Escherichia coli* (*E. coli*) TG1 (Novagen) was a general cloning host. *E. coli* ET12567/pUZ8002 and ET12567/pUB307 were used to introduce plasmid into *Streptomyces*. *E. coli* GB05RedTrfA (containing pSC101-BAD-ETgA-tet) was used to clone the daptomycin BGC (Wang et al., 2016).

Solid and shake-flask fermentation medium was described previously (Kieser et al., 2000; Wang et al., 2014). In fed-batch fermentation, the primary seed medium contained 3% Tryptic Soy Broth (TSB) and 3% maltodextrin (MD), abbreviated as TSB-MD. The secondary seed medium contained 6% maltodextrin, 2.5% soybean powder, 1.5% glucose, 0.08% (NH_4_)_2_Fe(SO_4_)_2_⋅6H_2_O, 0.1% Yeast extract, 0.5% calcium carbonate, and 0.2% molasses. The fermentation medium contained 7.2% maltodextrin, 1.2% yeast powder, 1.0% glucose, 0.08% (NH_4_)_2_Fe(SO_4_)_2_⋅6H_2_O, 0.72% molasses, and 0.1% defoamer GPE.

### Plasmid Construction

The plasmids and primers used in this article are shown in Supplementary Tables 1, 2, respectively. Primer pairs 1/2 and 3/4 were used to amplify the left and right homologous arms, which were cloned into pKC1139 to get the plasmid pKC1139-Δ*orf3244*. Similar to the above construction process, we got pKC1139-Δ*arpA* and pKC1139-Δ*orf3265-*Δ*orf3266*. Deletion of *orf3242* was implemented by using the CRISPR/Cpf1-mediated gene-editing system. The software sgRNA Scorer version 2.0 was used to design the spacer sequences, which target the deletion sequences with Protospacer Adjacent Motif (PAM) TTV [the PAM of Cpf1 where T is thymine and V is nucleotide A(adenine), C(cytidine), or G(guanine)] at its 5’ end (Chari et al., 2017). The primer pair 5/6 was used to amplify the CRISPR RNA (CrRNA) cassettes. Primer pairs 7/8 and 9/10 were used to amplify two homologous arms. Then, assembled homologous arms were obtained by overlapped PCR. Finally, the ClonExpress II One Step Cloning Kit (Vazyme Biotech Corporation, China) was used to connect *Nde*I/*Spe*I-digested pKCCpf1 and the overlapping PCR products to get plasmid pKCCpf1-Δ*orf3242*. Similar to the construction of plasmid pKCCpf1-Δ*orf3242*, we got pKCCpf1-Δ*phaR*. The primer pair 11/12 was used to amplify *dptJ*, which was cloned into pIJ8661, an integrative plasmid containing the strong promoter ermEp*, to get pIJ8661-*dptJ*. Primers 13 and 14 were used to amplify *orf3245* with its native ribosome binding site (RBS), which was cloned into pIJ8661-*dptJ* to get pIJ8661-*dptJ-orf3245*. Primers 15 and 16 were used to amplify *orf3243* with its native RBS, which was cloned into pIJ8661-*dptJ-orf3245* to get pIJ8661*-dptJ-orf3245-orf3343*. The plasmid pIJ8661*-dptJ-orf3245-orf3343* was digested by *Nde*I/*Kpn*I to get *Nde*I*-dptJ-orf3245-orf3343-Kpn*I, which was ligated to *Nde*I/*Kpn*I-digested pSOK804 by the T4 DNA ligase (Vazyme Biotech Corporation Ltd., China) to get plasmid pSOK804-ermEp*-*dptJ-orf3245-orf3343* at last. The primer pair 30/31 was used to amplify ampicillin (AMP) resistance gene fragment by PCR with the plasmid pUT18 as a template. The primer pair 32/33 was used to amplify the attP-integrase fragment with plasmid pSET152-*vgb2* as a template. The attP-φ31 integrase-AMP cassette was obtained by fusion PCR and then it was electroporated into arabinose-induced *E. coli* GB2005RedTrfA and inserted into plasmid pBeloBac*:*dpt* (Supplementary Table 1) by *in vivo* linear-circular homologous recombination (LCHR), generating the plasmid 701DIAA. The primer pair 34/35 was used to amplify homologous recombination fragment (-SmR-) containing spectinomycin resistance (*SmR*) gene and then it was inserted into 701DIAA by *in vivo* LCHR, generating the final plasmid 702DIAAS. Recombinant plasmids 701DIAA and 702DIAAS were identified by PCR (Supplementary Figure 2). The primer pair 36/37 was used to amplify *Vitreoscilla* hemoglobin (*vgb2*) gene with pSET152-*vgb2* as template and then the *vgb2* fragment was ligated to *Nde*I/*Kpn*I-digested pSOK804 by T4 DNA ligase. All the fragments were amplified with KOD (the trade name of DNA polymerase from the hyperthermophilic Archaeon *Thermococcus kodakaraensis* KOD1) plus-neo DNA polymerase (Toyobo), a high-fidelity DNA polymerase.

### Building of Metabolic Network Models Based on Genome

Genomic analyses of *S. roseosporus* L2790 were performed by the Rapid Annotations using Subsystems Technology (RAST) Server^1^ (Aziz et al., 2008). According to analysis results, the putative Kyn metabolic network model was built. In addition, we searched for genes perhaps participating in pigment synthesis in the Spreadsheet downloaded from the RAST Server.

### Construction and Fermentation of *Streptomyces roseosporus* Strains

The plasmid pKC1139-Δ*orf3244* was introduced into *S. roseosporus* L2790 by conjugation to get L2791. The deletion plasmid pKCCPf1-Δ*orf3242* was introduced into L2791 by conjugation to get L2792. Similarly, plasmid pKCCPf1-*phaR* and pKC1139-*arpA* were used to knock out gene *phaR* and *arpA* in sequence in L2792 to get L2795. The plasmid pKC1139-Δ*orf3265-*Δ*orf3266* was introduced into L2795 by conjugation to get L2796. The strains L2791, L2792, L2795, and L2796 were all obtained by using the in-frame deletion strategy. Furthermore, the plasmid pSOK804-*dptJ-sro3245-orf3343* was introduced into L2792 to get strain L2792a. Plasmid 702DIAAS was introduced into L2796 to get L2797. Plasmid pSOK804-*vgb2* was introduced into L2797 to get L2797-VHb, which can heterologously express VHb protein in L2797. Strains L2792a, L2797, and L2797-VHb were all obtained by insertion of target genes into the genome *via* site-specific integrase, respectively. All the recombinant strains were identified by PCR (Supplementary Figure 3). Shake-flask and scale-up fermentation were performed according to Supplementary Fermentation Process Document.

### Analysis of Daptomycin by High-Performance Liquid Chromatography

The C18 reverse-phase column (Zorbax 300SB-C18, 5 μm, 4.6 mm × 250 mm) from Agilent (Wang et al., 2014) was used to analyze daptomycin by high-performance liquid chromatography (HPLC) at a rate of 1 ml/min with mobile phases, including solution A (H_2_O containing 0.1% Formic acid) and solution B (acetonitrile). The HPLC procedure and samples preparation were described previously (Luo et al., 2018a). UV detection wavelength was 215 nm.

### Creation of Heatmap

Programs, such as Local Basic Local Alignment Search Tool (BLAST) and TBtools, were used to build a heatmap. In brief, EC 2.6.1.7 may be a kynurenine aminotransferase or kynurenine–oxoglutarate transaminase according to sequence data for EC numbers in the Kyoto Encyclopedia for Genes and Genomes (KEGG). In the National Center for Biotechnology Information (NCBI) database, we chose a kynurenine aminotransferase AM113472 from *Micromonospora* spp. *ML1* and a kynurenine–oxoglutarate transaminase NP_012475 from *Saccharomyces cerevisiae* S288C for local BLAST. In addition, an aspartate aminotransferase AAF10201 from *Deinococcus radiodurans* R1, which had the closest match with AM113472 (Lombo et al., 2006), was also chosen for local BLAST. A heatmap was built by using TBtools according to the local BLAST results.

### Analysis of Vitreous Hemoglobin by Carbon Monoxide-Difference Spectrum

Carbon monoxide (CO)-difference spectrum was used to detect the activity of VHb (Zhu et al., 2011; Li et al., 2016). The mycelia of L2797 and L2797-VHb were broken with an ultrasonic homogenizer (JY92-IIDN, Ningbo Scientz Biotechnology Corporation Ltd.) on ice for 15 min with a cycle of a 3-s work and a 5-s pause. The working power was 35%. The CO-difference spectrum was obtained by scanning in the 400–550 nm range using a UV-visible spectrophotometer (Evolution 220, Thermo Fisher Scientific Corporation Ltd.).

### Concentration Measurement of Tryptophan and Kynurenine by High-Performance Ligquid Chromatography

In shake-flask fermentation progress, 1 ml of culture was harvested each 24 h. The mycelia were washed and then disrupted by sonication with 500 μl phosphate buffer (50 mM, pH = 7.0). The content of total protein was determined by the Bradford method. Tryptophan and Kyn in the lysate were analyzed by HPLC (1260 Infinity, Agilent Technologies) with a reverse-phase column (Zorbax 300SB-C18, 5 μm, 4.6 mm × 250 mm; Agilent Technologies) with solution A (15 mM sodium acetate, pH = 4.0) and solution B (100% acetonitrile) at the ratio of A:B = 92:8. The detected wavelength was 360 nm for Kyn and 280 nm for tryptophan. The flow rate was 1.0 ml/min. 100 μl of cleared lysate was injected for HPLC analysis.

## Results and Discussion

### Improve Daptomycin Titer by Enhancing Kynurenine Supply

Kynurenine is a non-proteinogenic amino acid, which is an important precursor for the synthesis of daptomycin. Daptomycin production was improved by partially modifying the Kyn pathway (Liao et al., 2013). We measured the concentration of Kyn and tryptophan in the cytoplasm of strain L2790 (Supplementary Figure 4). The results showed that the concentration of tryptophan varied from 0.08 to 0.15 nmol/μg (amino acid/total protein), while the concentration of Kyn was 0.003 nmol/μg in 24 h and was too low to be detected in other time points. It suggested that the supply of Kyn was seriously inadequate or the kynurenine was drained out rapidly as soon as it was generated. Therefore, we attempted to systematically enhance the metabolic flux to supply more Kyn.

**FIGURE 2:**
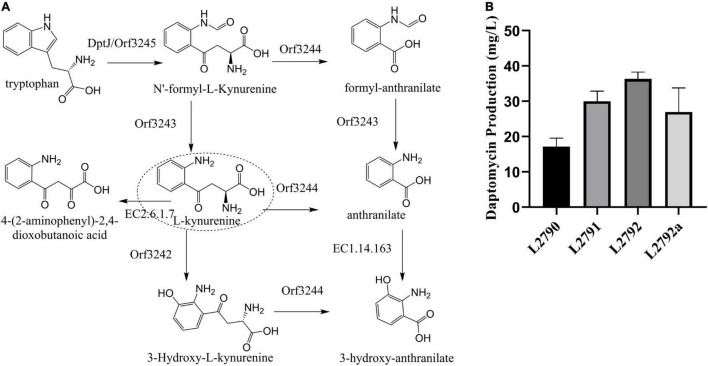
Improving daptomycin production by enhancing Kyn precursor supply. **(A)** The putative L-Kyn metabolic network in strain L2790 according to the RAST Server. DptJ/Orf3245, tryptophan-2,3-dioxygenase; Orf3244, L-kynureninase; Orf3243, N′-formyl-L-kynurenine formidase; Orf3242, oxidoreductase; EC 2.6.1.7, a putative kynureninase aminotransferase. **(B)** The daptomycin production of different strains obtained in a stepwise manner. The culture samples were collected at 144 h and daptomycin production was analyzed by high-performance liquid chromatography (HPLC). The fermentations were performed in triplicate. L2790, initial strain; L2791, the resultant strain by deleting *orf3244* in L2790; L2792, the resultant strain by deleting *orf3242* in L2791; L2792a, the resultant strain by co-overexpressing *dptJ*, *orf3245*, *orf3243* driven by ermEp* in L2792.

The L-Kyn metabolic network based on the KEGG metabolic analysis in the strain L2790 was established using the RAST Server (Figure 2A; Aziz et al., 2008). At least five proteins were found to be involved in its metabolic pathway (Supplementary Table 2). Among them, tryptophan-2,3-dioxygenase, including DptJ and Orf3245, converts tryptophan to N′-formyl-L-kynurenine (NFK). NFK was converted to Kyn by an NFK formamidase (Orf3243) or formylanthranilate by L-Kynureninase (Orf3244). L-Kyn can be turned to anthranilate catalyzed by Orf3244, 3-hydroxy-L-kynurenine by an oxidoreductase (Orf3242), or 4-(2-aminophenyl)-2,4-dioxobutanoate by a kynurenine aminotransferase (EC2.6.1.7), which may be one of three putative proteins, namely, Orf3256, Orf2688, or Orf2371, according to a heatmap (Supplementary Figure 5).

To improve Kyn supply, we firstly knocked out *orf3244* and *orf3242* in turn. Disruption of *orf3244* in L2790 got strain L2791, in which daptomycin concentration reached 30.0 mg/l with an increase of 74% compared with L2790 (Figure 2B). Disruption of *orf3242* in L2791, generating strain L2792, further improved daptomycin production to 36.3 mg/l, 1.21-fold of strain L2791 (Figure 2B). Besides, gene *dptJ*, *orf3245*, and *orf3243* were involved in kynurenine biosynthesis according to L-Kyn metabolic network (Figure 2A). Therefore, we co-overexpressed *dptJ*, *orf3245*, and *orf3243* under the control of a strong promoter ermEp* in L2792, generating strain L2792a. However, daptomycin production of L2792a did not increase (Figure 2B).

### Engineering of Regulatory Pathway in Daptomycin Biosynthesis

**FIGURE 3:**
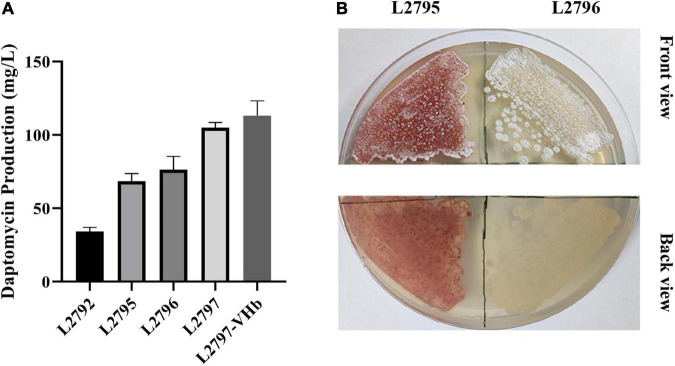
Improving daptomycin production and quality by regulatory pathway reconstruction, pigment removal, multicopy BGC, and heterologous expression of VHb in a stepwise manner. **(A)** The daptomycin production of different strains obtained in a stepwise manner. The culture samples were collected at 144 h and daptomycin production was analyzed by HPLC. The cultures were performed in triplicate. L2792, the resultant strain by deleting *orf3244* and *orf3242* in the initial strain L2790; L2795, the resultant strain by deleting gene *phaR and arpA* in L2792; L2796, the resultant strain by deleting gene *orf3265 and orf3266* in L2795; L2797, the resultant strain by introducing the extra copy of daptomycin BGC into L2796; L2797-VHb, the resultant strain by introducing a *vgb2* gene into L2797. **(B)** Phenotype of L2795 and L2796 cultured on R5 medium.

ArpA and PhaR are two known pleiotropic regulators in *S. roseosporus*. ArpA indirectly and negatively regulates the transcription of daptomycin BGC through the A factor signaling pathway, while PhaR direct negatively regulates the transcription level of the gene cluster by binding to the transcription initiation region of the daptomycin BGC (Mao et al., 2015; Luo et al., 2018b). Thus, these two regulatory genes were knocked out to remove their negative effect on daptomycin biosynthesis. The resultant strain L2795 produced 68 mg/l daptomycin, an increase of 90%, compared with L2792 (Figure 3A).

### Blocking the Red Pigment Synthesis to Eliminate the Byproduct in *Streptomyces roseosporus*

Red pigment is observed as the main byproduct accompanied by the daptomycin production *in S. roseosporus* L2795 (Figure 3B), which seriously affected the following separation and purification process, thereby reducing daptomycin quality and increasing the production cost. The RAST Server was used to find the pathway of pigment biosynthesis and five genes could be involved in the synthesis of the pigment (Supplementary Table 2). Among them, *orf5781*, a putative indigoidine synthase A-like protein-encoding gene, might not be related to red pigment synthesis and its loci were also far away from the other four genes, namely, *orf3259*, *orf3265*, *orf3266*, and *orf3267* (Supplementary Table 2). According to the gene function annotated by the RAST Server, these four genes are involved in red pigment biosynthesis. Orf3259 is polyketide cyclase WhiE VII, Orf3265 is polyketide chain length factor WhiE-CLF, Orf3266 is polyketide beta-ketoacyl synthase WhiE-KS, and Orf3267 is polyketide cyclase WhiE II. They are all the typical type II PKS and adjacent to each other. It is reported that many pigment-type natural products are synthesized by type II PKS (Staunton and Weissman, 2001). To prove this idea, *orf3265* and *orf3266* were knocked out to block the synthesis of red pigment, generating strain L2796. Compared with the parent strain L2795, the aerial mycelium of L2796 turned white (Figure 3B) and, meanwhile, the fermentation titer of daptomycin in shake flasks was increased by 10% (Figure 3A), indicating that deletion of byproduct pathway may reduce the metabolic burden and facilitate the production of target daptomycin.

### Improving Daptomycin Production by Duplication of *dpt* Biosynthetic Gene Cluster

Increasing the copy number of BGC is an effective way to increase the yield of natural products (Li et al., 2021c). A previous study showed that daptomycin BGC with only 65 kb length was enough to be heterologously expressed in daptomycin non-producing strain *S. coelicolor* M511 and successfully led to the production of 28.9 mg/l daptomycin (Choi et al., 2019). pBeloBac*:*dpt* is a plasmid containing a large size of daptomycin BGC of 128 kb. We first reduced its size by removing redundant genes (spanning about 60 kb) to decrease the metabolic burden and increase the efficiency of conjugation into hosts. We tried to simplify the plasmid by LCHR. The *dptR1* gene locates downstream in daptomycin BGC. A previous study showed that daptomycin production decreased no matter what *dptR1* was deleted or overexpressed (Yu et al., 2020). Therefore, the simplification of the downstream gene sequence started from *dptR1* and, meanwhile, integrase φ31 and AMP-resistant gene were introduced by cassette attP-φ31 integrase-AMP. The upstream *dptP* gene of the *dpt* BGC is benefit to daptomycin resistance (Zhang et al., 2020). Therefore, we select a suitable position (near gene *orf6577*) upstream of the *dptP* gene as the starting position to simplify the upstream gene sequence. Linear recombination fragment—SmR—was used to simplify the upstream region of the daptomycin BGC. Finally, the resulting plasmid 702DIAAS was introduced into L2796 to get L2797. The production of daptomycin in L2797 reached 105 mg/l, an increase of 40%, compared with the parental strain L2796 (Figure 3A).

### Fermentation Process Engineering to Enhance Daptomycin Biosynthesis

**FIGURE 4:**
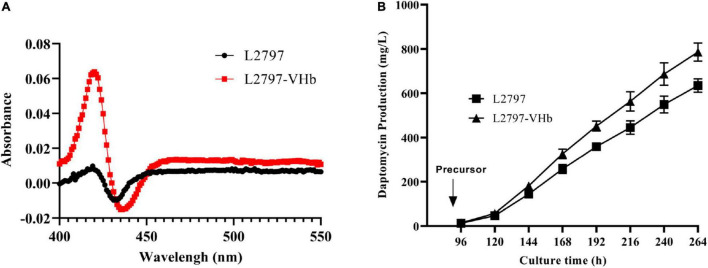
Improving daptomycin production by fermentation process engineering. **(A)** Carbon monoxide (CO)-difference spectra analysis of L2797-VHb and L2797. **(B)** The daptomycin production curves of L2797-VHb and L2797 during the fermentation process in the 15-L fermenter. The fermentation samples were collected every 24 h from 96 to 264 h and the daptomycin production was analyzed by HPLC. The fermentation for each strain was performed in triplicate. Addition of precursor started from 96 h.

To test the ability of L2797 to produce daptomycin in the fermenter, we conducted fed-batch fermentation in a 15-L fermenter with 9 L working volume. The production of daptomycin reached 635 mg/l. However, throughout the fermentation process, although the stirring speed has been gradually increased to a maximum speed of 450 rpm, the dissolved oxygen value still often touched around 0. Enough oxygen supply is critical for energy metabolism, primary metabolite, and secondary metabolite production. Even though higher stirring speed may ensure sufficient oxygen, the filamentous differentiation of some *Streptomyces*, such as *S. roseosporus*, will be damaged under high stirring shear force, which is pivotal for the secondary metabolite production with high titer (Ferraiuolo et al., 2021). Hence, we try to express heterologous *Vitreoscilla hemoglobin* (VHb) protein in *S. roseosporus* to enhance the utilization efficiency of oxygen under low dissolved oxygen conditions to improve daptomycin production. According to the results of the CO-difference spectrum absorbance assay, an obvious absorption peak at 420 nm was observed in strain L2797-VHb compared with L2797 (Figure 4A). These results showed that the recombinant strain L2797-VHb expressed functional VHb successfully. The growth curve indicated that L2797-VHb exhibited better oxygen utilization efficiency than L2797 under simulated low dissolved oxygen state (Supplementary Figure 6). As we expected, the daptomycin titer of L2797-VHb was further increased to 113 mg/l under normal conditions described previously in shake flasks (Figure 3A). In the fed-batch fermentation of 15 L fermenter, the ability of the resultant strain L2797-VHb to produce daptomycin reached as high as 786 mg/l, an increase of 24%, compared with the parent strain L2797 (Figure 4B).

## Discussion

Various metabolic engineering strategies have been developed to improve the production of natural products (Bu et al., 2021). As one of the natural products, commercially available daptomycin (e.g., Cubicin) is widely used in anti-inflammation, skin infection, and bacteremia, etc., bringing huge market demands for this antibiotic. The global market size of daptomycin is expected to be 5.1 billion USD and is expected to register a growth of 4.4% from 2020 to 2027 (Market Research Future, 2021). Although many efforts have been made to enhance the yield of daptomycin for cost reduction, such as refactoring regulatory pathway, enhancing precursor supply, or traditional mutagenesis, most of these strategies are clearly targeted at a single factor, which cannot improve the production of daptomycin at global or systematic level (Supplementary Table 4). The daptomycin fermentation titer remains deficient or the red pigment is not removed. Combined with our previous studies on daptomycin biosynthesis (Mao et al., 2015; Luo et al., 2018b), we designed a combinatorial engineering strategy to further advance the biosynthetic efficiency of daptomycin.

With the help of genomic analysis, we first built a Kyn metabolic network model. According to the model, we improved daptomycin production to 36.3 mg/l with an increase of 111% by knocking out kynurenine-related genes, such as *orf3344* and *orf3242* (Figure 2B). However, co-overexpression of other related genes, such as *dptJ*, *orf3245*, and *orf3243*, made daptomycin production decrease by 26% (Figure 2B) and this may be due to the excessive accumulation of some intermediates, which affect the further synthesis of L-Kyn (Ji et al., 2021) or are toxic to the cell itself, thereby affecting the accumulation of daptomycin. The underlying mechanisms may be complicated. Like Kyn, decanoic acid is also an important precursor substance in the biosynthesis of daptomycin. Excess decanoic acid is toxic to cells (Lee et al., 2016). Therefore, during the fed-batch fermentation process in a 15 L fermenter, we used a constant rate to add decanoic acid to keep its concentration at a low level in the culture. In addition, during the shake-flask fermentation process, we found that the growth of the bacteria was stagnant and it was difficult to enter the secondary metabolism if we added decanoic acid too early, such as cultivating at 24 h (data not shown). Therefore, we speculate that enzymes involved in the metabolism of decanoic acid may only gradually begin to be expressed during the transition from primary metabolism to secondary metabolism. If we compare and analyze the transcriptome before and after the addition of capric acid, we may be able to find which enzymes are mainly involved in its metabolism and transport and improve the tolerance and utilization efficiency of decanoic acid by modifying these enzymes.

On the basis of refactoring the Kyn pathway, we deleted two negative regulators, namely, ArpA and PhaR, at the same time, which made daptomycin titer increase by 88% and reach 68 mg/l (Figure 3A). Although knocking out *arpA* or *phaR* can increase the production of daptomycin, they played different roles in the growth and differentiation of hyphae (Mao et al., 2015; Luo et al., 2018b). Whether there is a cross-talking effect mechanism from both the regulatory genes on mycelial growth and daptomycin accumulation, it could be further investigated through transcriptome analysis. There may exist a sophisticated and cascaded regulatory network, which can facilitate us to fine-tune it for further improvement of daptomycin production.

Red pigment is a visible impurity in the production of daptomycin. Usually, many pigment biosynthetic pathways belong to type II PKSs in *Streptomyces*. Short-chain acyl-CoAs, such as malonyl-CoA, methylmalonyl-CoA, and ethylmalonyl-CoA, are important precursors of many type II PKS pathways (Lin et al., 2020; Bednarz et al., 2021). Acetyl-CoAs are the main elements for the formation of short-chain acyl-CoA precursor pool. As we all know, acetyl-CoAs also participate in primary metabolisms, especially the tricarboxylic acid (TCA) cycle, for the supply of energy responsible for cell growth and primary/secondary metabolisms, such as gene expression, amino acid synthesis, and natural product biosynthesis. Here, amino acid synthesis is pivotal for daptomycin biosynthesis because many amino acids are direct precursors of daptomycin. Therefore, disruption of pigment biosynthesis can block the consumption of short-chain acyl-CoA precursors and enrich more acetyl-CoAs toward primary metabolism, thereby supplying more energy or precursors (especially amino acids) for daptomycin production. Besides, pigment production will negatively affect the purification of target metabolites. For example, in the production of amphotericin B, a ceramic membrane is usually used to remove pigments (Kai et al., 2020). During the extraction of daptomycin, we also observed that a large number of red pigments are accompanied. Furthermore, the biological activity of red pigments also remains unknown and it is required to remove them for the purity of commercial daptomycin. We next removed it in strain L2795 obtained by enhancing the Kyn pathway and deleting two negative regulators. The daptomycin titer of the resulting strain L2796 reached 76 mg/l with an increase of 10% and its red pigment was removed thoroughly (Figures 3A,B). In addition to the red pigment, the production process of daptomycin is also accompanied by other byproducts, such as homologs of daptomycin. Although deleting branched-chain α-keto acid dehydrogenase (BKD) enzyme complex could remove homologs of daptomycin containing branched-chain fatty acids (BCFAs), it introduced new homologs of daptomycin with straight-chain fatty acids (Ji et al., 2021). Therefore, in future studies, other methods should be considered to completely solve the problem of daptomycin homologs, such as the directed evolution of DptE, an acyl AMP ligase, on improving its substrate selectivity. Besides, according to the anti-SMASH annotation of genome, there are other 27 non-target BGCs, except daptomycin BGC, which may compete precursor and/or energy flux with daptomycin pathway and result in metabolic burden. Transcriptome can be conducted to determine the active BGCs and then we can delete them to enrich metabolic flux toward the daptomycin pathway and, meanwhile, simplify metabolic background for the improvement of daptomycin production in yield and quality.

Finally, we further increased daptomycin to 105 mg/l with an increase of 40% by introducing an extra copy of daptomycin BGC, which implied that multicopy of BGC can efficiently enhance the biosynthesis of daptomycin (Figure 3A). According to previous studies, the overall transcriptional level of dpt BGC is too low to meet the efficient production of daptomycin in *S. roseosporus* (Ji et al., 2021). Recently, Lu’s team developed an integrase-mediated multicopy system used for the integration of 3–5 copies BGCs into *Streptomyces* genomes. For example, the start and engineered 5-oxomilbemycin-producing strains, such as KF200, KF201, KF202, and KF203, harboring 1–4 copies of 5-oxomilbemycin BGCs by advanced Multiplex Site-specific Genome Engineering (aMSGE) system, respectively, can make a stepwise increase in 5-oxomilbemycin production (from 2,228, 4,415, 5,592 to 6,368 mg/l; Li et al., 2019). Previous studies also suggested that tandem amplification of BGCs could increase the copy number (4–12 copies), thereby enhancing the production of natural products, such as actinorhodin, bleomycin, and validamycin A (Murakami et al., 2011; Zhou et al., 2014, Li et al., 2021a,b). These studies strongly imply that increasing the cluster copies can further enhance the production of target metabolites. From the perspective of gene expression, multicopy is actually equivalent to overexpressing every gene in the BGC and the former is a more efficient strategy than the latter because upregulating all the genes needs to design different promoters to balance their expressions for avoiding toxic intermediates or incomplete conversion. Of course, the yields of metabolites may depend more on the tolerance of engineered strains to specialized metabolites. In the future, we will first investigate the resistance level of *S. roseosporus* against daptomycin and then determine the possible copy number of *dpt* BGC or upregulate the expression of transporters to increase the tolerance of engineered strains for the maximized production of daptomycin. We can also effectively combine the above engineering strategies to obtain a high-performance cell factory with higher daptomycin production in future studies.

Here, we make a comparison among different engineering strategies for the improvement of daptomycin production (Supplementary Table 4). We can see that our strategy not only remove red pigment but also efficiently enhance the production of daptomycin, which reached as high as 786 mg/l in the 15 L fermenter. Although the addition of sodium decanoate can substantially increase the yield of daptomycin sharply, it is a surfactant. In our shake-flask experiments, adding sodium decanoate to the fermentation broth resulted in a lot of foam, which may cause the fermentation broth to escape and become contaminated during large-scale fermentation. Therefore, we chose decanoic acid as the feed precursor instead of sodium decanoate. However, in the future, we can try to explore a new process that can greatly increase the production of daptomycin through sodium caprate and eliminate the generation of foam at the same time.

**FIGURE 5:**
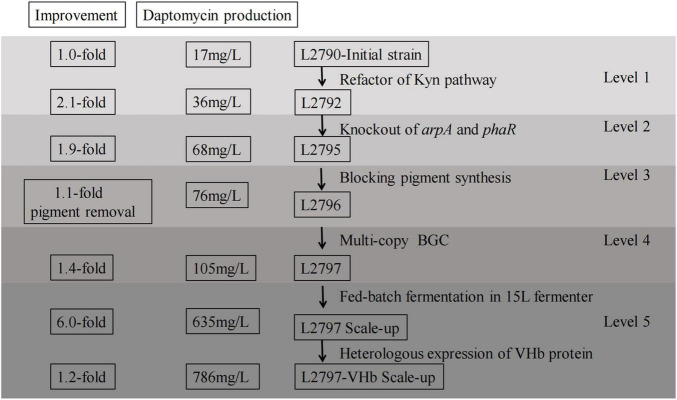
Diagram summarizing multilevel metabolic engineering strategies implemented to daptomycin production and quality. Counting from the right, the first column, the level number for each metabolic engineering approach; the second column, the specific implementation content for each step of combinatorial metabolic engineering strategies from the beginning to the end; the third column, daptomycin production caused by each metabolic engineering approach; the last column, the corresponding fold changes caused by each metabolic engineering approach.

In conclusion, this study revealed an integrated metabolic engineering strategy for daptomycin production in *S. roseosporus* for the first time. Step by step, the combinatorial approach was employed to create daptomycin overproducing strains (Figure 5). Daptomycin production was increased by roughly 5-fold using rational design and refactoring at the molecular level and an additional about 7-fold using fermentation process engineering. This study not only boosts daptomycin productivity but also enhances its quality with low byproducts. Importantly, the engineering strategies used here are expected to significantly reduce expenditure for daptomycin industrial manufacturing.

## Data Availability Statement

The original contributions presented in this study are included in the article/Supplementary Material, further inquiries can be directed to the corresponding author.

## Author Contributions

Z-YL contributed to conceptualization, methodology, validation, investigation, data curation, formal analysis, visualization, writing—original draft, and writing, reviewing and editing the manuscript. Q-TB and J-LF contributed to methodology, data curation, formal analysis, software, 15 L fermentation, and writing, reviewing and editing the manuscript. C-YZ and W-FX contributed to methodology, data curation, and 15 L fermentation. LM, W-LG, and X-AC contributed to methodology, data curation, software, and writing, reviewing and editing the manuscript. Y-QL contributed to conceptualization, project administration, funding acquisition, supervision, and writing, reviewing, and editing the manuscript. All authors read and approved final version of the manuscript.

## Conflict of Interest

The authors declare that the research was conducted in the absence of any commercial or financial relationships that could be construed as a potential conflict of interest.

## Publisher’s Note

All claims expressed in this article are solely those of the authors and do not necessarily represent those of their affiliated organizations, or those of the publisher, the editors and the reviewers. Any product that may be evaluated in this article, or claim that may be made by its manufacturer, is not guaranteed or endorsed by the publisher.

## Footnotes

1
https://rast.nmpdr.org/
