# Supplementary material for: Improving the Yield and Quality of Daptomycin in Streptomyces roseosporus by Multilevel Metabolic Engineering
Source: Front Microbiol. 2022 Apr 18;13:872397. doi: 10.3389/fmicb.2022.872397 (PMC9058172; doi:10.3389/fmicb.2022.872397)
Supplement: Supplementary file 1 [file Table_1.DOCX]

**Table S1** Plasmids and strains used in this study

| **Plasmids or strains** | **Description** | **Reference or source** |
| --- | --- | --- |
| **Plasmids** |  |  |
| pKC1139 | temperature-sensitive shuttle vector for gene knock-out in Streptomyces | (Bierman et al., 1992) |
| pKCCpf1 | A vector derived from pKC1139 containing the *scocpf1* gene under the control of *ermEp** and the crRNA repeat unit under the control of *kasOp** | Professor Yinhua Lu’s lab |
| pKC1139-*Δorf3244* | *orf3244* deletion plasmid | This study |
| pKCCpf1*-Δorf3242* | *orf3242* deletion plasmid | This study |
| pIJ8661 | overexpression vector containing a strong promoter *ermEp**, integrative in *Streptomyces* (φC31 integrase system) | (Liu et al., 2015) |
| pIJ8661-*dptJ* | *dptJ* overexpression plasmid | This study |
| pIJ8661-*dptJ-orf3245* | *dptJ* and *orf3245* co-overexpression plasmid | This study |
| pIJ8661-*dptJ-orf3245-orf3243* | *dptJ*, *orf3245* and *orf3243* co-overexpression plasmid | This study |
| pSOK804 | overexpression vector containing a strong promoter *ermEp**, integrative in *Streptomyces* (VWB integrase system ) | Professor Wen Liu’s lab |
| pSOK804-*dptJ-sro3245-orf3343* | *dptJ*, *orf3245* and *orf3243* co-overexpression plasmid | This study |
| pKC1139-*ΔarpA* | *arpA* deletion plasmid | (Mao et al., 2015) |
| pKC1139-*Δorf3265-Δorf3266* | *orf3265* and *orf3266*deletion plasmid | This study |
| pKCCpf1-*ΔphaR* | *phaR* deletion plasmid | This study |
| pUT18 | As a template for amplifying AMP gene fragment by PCR | Euromedex |
| pBeloBac*::dpt | A plasmid containing the entire *dpt*-gene cluster and the oriT-apramycin cassette | Professor Huarong Tan’s lab |
| 701DIAA | A recombinant plasmid containing the entire *dpt*-gene cluster and attP-integrase-AMP, oriT-apramycin cassettes | This study |
| 702DIAAS | A recombinant plasmid containing a simplified *dpt*-gene cluster and Spectinomycin, attP-integrase-AMP, oriT-apramycin cassettes | This study |
| pSET152-*vgb2* | *vgb2* overexpression plasmid | Professor Cunjiang Song’s lab |
| pSOK804-*vgb2* | *vgb2* overexpression plasmid | This study |
|  |  |  |
| **Strains** |  |  |
| ***Escherichia coli*** |  |  |
| TG1 | General cloning host | Invitrogen |
| ET12567/pUB307 | ET12567 containing the transmissible RP4 derivative plasmid pUB307; helper strain for intergeneric conjugation; | Invitrogen |
| ET12567/pUZ8002 | ET12567 containing the non-transmissible RP4 derivative plasmid pUZ8002; helper strain for intergeneric conjugation; | Invitrogen |
| GB05RedTrfA | DH10B, *fhuA*::IS2, Δ*ybcC*, Δ*recET*, P_RhaSR_-*γβαA,* P_BAD_-*trfA* | Professor Youming Zhang’s lab |
|  |  |  |
| *Streptomyces roseosporus* |  |  |
| L2790 | A daptomycin-producing strain | Our lab |
| L2791 | L2790 mutant (Δ*orf3244*) | This study |
| L2792 | L2790 mutant (Δ*orf3244,* Δ*orf3242*) | This study |
| L2792a | L2792 containing the plasmid pSOK804-*dptJ-sro3245-orf3343* | This study |
| L2795 | L2790 mutant (Δ*orf3244,* Δ*orf3242,* Δ*phaR,* Δ*arpA*) | This study |
| L2796 | L2790 mutant (Δ*orf3244,* Δ*orf3242,* Δ*phaR,* Δ*arpA,*  *Δorf3265, Δorf3266*) | This study |
| L2797 | L2796 containing the plasmid 702DIAAS | This study |
| L2797-VHb | L2797 containing the plasmid pSOK804-*vgb2* | This study |

**Supplemental Material References**

Bierman, M., Logan, R., O'Brien, K., Seno, E.T., Rao, R.N., Schoner, B.E. (1992). Plasmid cloning vectors for the conjugal transfer of DNA from *Escherichia coli* to *Streptomyces spp*. *Gene* **116**(1), 43-49. doi: 10.1016/0378-1119(92)90627-2.

Liu, S.P., Yuan, P.H., Wang, Y.Y., Liu, X.F., Zhou, Z.X., Bu, Q.T., et al. (2015). Generation of the natamycin analogs by gene engineering of natamycin biosynthetic genes in *Streptomyces chattanoogensis* L10. *Microbiol. Res.* **173**, 25-33. doi: 10.1016/j.micres.2015.01.013.

Mao, X.M., Luo, S., Zhou, R.C., Wang, F., Yu, P., Sun, N., et al. (2015). Transcriptional regulation of the daptomycin gene cluster in *Streptomyces roseosporus* by an autoregulator, AtrA. *J. Biol. Chem.* **290**(12), 7992-8001. doi: 10.1074/jbc.M114.608273.
