# Supplementary material for: Improving the Yield and Quality of Daptomycin in Streptomyces roseosporus by Multilevel Metabolic Engineering
Source: Front Microbiol. 2022 Apr 18;13:872397. doi: 10.3389/fmicb.2022.872397 (PMC9058172; doi:10.3389/fmicb.2022.872397)
Supplement: Supplementary file 2 [file Table_2.DOCX]

**Table S2** Gene annotation in *S. roseosporus* L2790

| **Module** | **Name of gene** | **Position (start-end)** | **Proposed function** |
| --- | --- | --- | --- |
| **KYN pathway Associated Genes** | [*orf3*](http://rast.nmpdr.org/seedviewer.cgi?page=Annotation&feature=fig\|6666666.290734.peg.6139)*242* | 3694181-3693336 | Oxidoreductase |
|  | [*orf3*](http://rast.nmpdr.org/seedviewer.cgi?page=Annotation&feature=fig\|6666666.290734.peg.6140)*243* | 3695212-3694289 | putative lipase/esterase |
|  | [*orf3*](http://rast.nmpdr.org/seedviewer.cgi?page=Annotation&feature=fig\|6666666.290734.peg.6141)*244* | 3696609-3695377 | Kynureninase (EC 3.7.1.3) |
|  | [*orf3*](http://rast.nmpdr.org/seedviewer.cgi?page=Annotation&feature=fig\|6666666.290734.peg.6142)*245* | 3697450-3696602 | Tryptophan 2,3-dioxygenase (EC 1.13.11.11) |
|  | [*orf6*](http://rast.nmpdr.org/seedviewer.cgi?page=Annotation&feature=fig\|6666666.290734.peg.6142)*598 (dptJ)* | 7389493-7390230 | Tryptophan 2,3-dioxygenase (EC 1.13.11.11) |
| **Pigment Synthesis Associated Genes** | [*orf32*](http://rast.nmpdr.org/seedviewer.cgi?page=Annotation&feature=fig\|6666666.290734.peg.349)*59* | 3710851_3711273 | Polyketide cyclase WhiE VII |
|  | [*orf32*](http://rast.nmpdr.org/seedviewer.cgi?page=Annotation&feature=fig\|6666666.290734.peg.350)*65* | 3721031_3719799 | Polyketide chain length factor WhiE-CLF |
|  | [*orf32*](http://rast.nmpdr.org/seedviewer.cgi?page=Annotation&feature=fig\|6666666.290734.peg.351)*66* | 3722329_3721046 | Polyketide beta-ketoacyl synthase WhiE-KS |
|  | [*orf32*](http://rast.nmpdr.org/seedviewer.cgi?page=Annotation&feature=fig\|6666666.290734.peg.351)*67* | 3723207_3722326 | Polyketide cyclase WhiE II |
|  | [*orf5*](http://rast.nmpdr.org/seedviewer.cgi?page=Annotation&feature=fig\|6666666.290734.peg.351)*781* | 6435239_6434352 | Indigoidine synthase A-like protein, uncharacterized enzyme involved in pigment biosynthesis |
