# Supplementary material for: Improving the Yield and Quality of Daptomycin in Streptomyces roseosporus by Multilevel Metabolic Engineering
Source: Front Microbiol. 2022 Apr 18;13:872397. doi: 10.3389/fmicb.2022.872397 (PMC9058172; doi:10.3389/fmicb.2022.872397)
Supplement: Supplementary file 3 [file Table_3.DOCX]

**Table S3** Primers used in this paper.

| No. | Sequence (5’-3’) |
| --- | --- |
| 1 | AAGCTTAGGTGATGAGGTGCAGCCCG |
| 2 | TCTAGAGGCTTCACCCCGCTGTACGT |
| 3 | GGATCCCGAGTTGCCGTCGAGGTAGA |
| 4 | GATATCTCCATCCACGAGAACCTGCT |
| 5 | CGAACTCCTGGTAGATGGACATATGTGGATCCTACCAACCGGCACGATT |
| 6 | CGTCGGCCCCGCTGGAGACCGGTGACGAATCTACAACAGTAGAAATTTGGCCA |
| 7 | ATTCGTCACCGGTCTCCAGCGGGGCCGACGATCAGGACCCGGATG |
| 8 | TGTTGCCGCTCAGACGGACCCTGAAAGCGCTGCTGA |
| 9 | TCAGCAGCGCTTTCAGGGTCCGTCTGAGCGGCAACA |
| 10 | TAATCAAGGGTACCTACGAGATATCGGGCACCTGACGCAGGCA |
| 11 | CATATGACAGCGCAGGACACCCG |
| 12 | GATATCGGGCGGTGCCGTCAG |
| 13 | GATATCGGAAGCTCGTGTCGAGGAG |
| 14 | TCTAGAGTCGCGGTCGTGGTCTCAG |
| 15 | TCTAGACAGGCCACGGGCAGAGGACGGTTG |
| 16 | TCTAGATCACCAGGCGAGCTGGGTGATCTC |
| 17 | ACTTCAAGCTTGATGCCGTTGGTGGACCC |
| 18 | ACTTAGGATCCAGCCTGCTTCGCCATTTC |
| 19 | ACTTAGGATCCGCTGCCTGATCCTCGACC |
| 20 | ACTTAGAATTCCTCCTCGGCACGCTGTTC |
| 21 | GGCTGCAGGTCGACTCTAGAGTCCTCGCTCCGCTATCTGCTC |
| 22 | CACGCATCGCTGTCCACCCT |
| 23 | GGACAGCGATGCGTGTCACCGTCCTCGATGAGCCG |
| 24 | TATGACATGATTACGATATCCCACCTTCCGGCTCACCAT |
| 25 | CCCGCGTCGCCTCGGTCATGCGAGTGTCATCTACAACAGTAGAAATTTGGCCA |
| 26 | AGATGACACTCGCATGACCGAGGCGACGCGGGTCGTAGCCGAAGA |
| 27 | GTGAGTGAGCGCACAACGTGATGTCAGCCTGGGGTTGC |
| 28 | CGTTGTGCGCTCACTCACCAGATT |
| 29 | TACCTACGAGATATCGACGCACTAGTTCGTGGAAGGCGTGGGAGGA |
| 30 | ATGATCAGATCACGGACGGCGACCGGGAGTTGCTGACGACGCGGAACCCCTATTTGTTT |
| 31 | GGAAGACGTAGCGGCGTAGGTCTGACGCTCAGTGGAACG |
| 32 | CGTTCCACTGAGCGTCAGACCTACGCCGCTACGTCTTCC |
| 33 | TCCTCCGAGACCCTCAACTTCGTCCTGGTCGACTTCAAGGGCCCGTACTGACGGACACA |
| 34 | ATGACACTCTCCAACGGCACCTTCGACCTGATCCTGGAATGTAGGCTGGAGCTGCTTCG |
| 35 | CACCACGATCAGCACGAGGATGTTGACGACGAGCTGTATAGTTCGTACAGTCTATGCCT |
| 36 | GGAATTCCATATGCTCGACCAGCAGACCA |
| 37 | CGGGGTACCTCACTCCACGGCCTGGG |
| 38 | TGGACACCGATGTCGAGCTG |
| 39 | CAATCGGAATCGCGTCCATGA |
| 40 | AATAAGGGCGACACGGAAAT |
| 41 | ACGAACGCCCTGGATCACC |
| 42 | GCGCTTGCTGCTTGGATG |
| 43 | AGTTCAGCAGATGGGCGTC |
| 44 | GCCCTGCTGCGTAACATC |
| 45 | CCGGAGCGTGAAGCTGTT |
| 46 | AGCGGGGCGAAGTACTCT |
| 47 | GAAGGTGACGGCAGGCACAC |
| 48 | GGTCGCCTCGATGCCGTT |
| 49 | GCGGATGGAGTTCCTGCT |
| 50 | CAGCTTGGAACGCTGCTG |
| 51 | GATATCGGGCGGTGCCGTCAG |
| 52 | CCGACACGCATTTCGAGGACC |
| 53 | TCGATCACATCGTCCTCGTG |
| 54 | CCACGATGACGAACAGCCACA |
| 55 | CCATCCGGCCCACCCGTA |
| 56 | CGTTCGTCCTCTTCGCCTCG |
| 57 | GGTGAACTGACCGCCATGCTC |
| 58 | CTCCTGCTCCAGGCAAAG |
| 59 | GGACACGATGCCAACACG |
| 60 | CGCTTCGGCAAGCTCAC |
| 61 | GTTCCACTGAGCGTCAGAC |
| 62 | ATGCTCGACCAGCAGAC |
| 63 | ATTTCACACAGGAAACAGC |
