# Supplementary material for: Improving the Yield and Quality of Daptomycin in Streptomyces roseosporus by Multilevel Metabolic Engineering
Source: Front Microbiol. 2022 Apr 18;13:872397. doi: 10.3389/fmicb.2022.872397 (PMC9058172; doi:10.3389/fmicb.2022.872397)
Supplement: Supplementary file 4 [file Table_4.DOCX]

**Table S4** **Comparison in terms of daptomycin yield**

| **Strategies** | **Yield（mg/L）** | **Improvement** | **Culturing vessel** | **References** |
| --- | --- | --- | --- | --- |
| Multil-level metabolic engineering | 113  (Without red pigment) | 6.1-fold  (Removing pigment impurity) | Shake flask | This study |
|  | 786 | 6.9-fold | 15-L Bioreactor |  |
| Deletion of *wblA* | ~ 40 | 1.5-fold | Shake flask | (Huang et al., 2017) |
| Deleting *depR2* gene | 50.8 | 2.5-fold | Shake flasks | (Mao et al., 2017) |
| Overexpression of *dptJ* | ~25 | 2.1-fold | Shake flasks | (Liao et al., 2013) |
| Deleting *kyn* gene | ~20 | 1.3-fold |  |  |
| Deleting *phaR* gene | 89.5 | 6.1-fold | Shake flask | (Luo et al., 2018) |
| Increasing DA resistance | ~ 40.5 | 1.4-fold | Shake flask | (Lee et al., 2016) |
| Heterologous expression of  *dpt* BGC in *S. lividans* | 55 (A21978C) | NA | Shake flask | (Penn et al., 2006) |
| Heterologous expression of  *dpt* BGC in *S. coelicolor M511* | 28.9 | NA | Shake flask | (Choi et al., 2019) |
| Adding sodium decanoate | 600 | 71.6-fold | 3.6-L Bioreactor | (Ng et al., 2014) |
| Adding cofactors | 632 | 4.5-fold | 7.5-L Bioreactor | (Yu et al., 2011b) |
| Radiation mutagenesis | 296 (A21978C) | 2.5-fold | 7.0-L Bioreactor | (Lu et al., 2011) |
| Compound mutagenesis | 616 | 6-fold | 7.5-L Bioreactor | (Yu et al., 2011a) |
| Overexpression of *depR1* | 474 | 1.4-fold | 50-L Bioreactor | (Yuan et al., 2016) |
| In silico aided metabolic engineering | 581.5 | 1.4-fold | 7.5-L Bioreactor | (Huang et al., 2012) |
| Genome shuffling | 582 | 4.8-fold | 7.5-L Bioreactor | (Yu et al., 2014) |

NA: Not Available; A21978C: daptomycin and its homologies; DA: decanoic acid.

References

Choi, S., Nah, H.J., Choi, S., Kim, E.S. (2019). Heterologous Expression of Daptomycin Biosynthetic Gene Cluster Via Streptomyces Artificial Chromosome Vector System. *J Microbiol Biotechnol*, **29**(12), 1931-1937. doi: 10.4014/jmb.1909.09022.

Huang, D., Wen, J., Wang, G., Yu, G., Jia, X., Chen, Y. (2012). In silico aided metabolic engineering of Streptomyces roseosporus for daptomycin yield improvement. *Applied Microbiology & Biotechnology*, **94**(3), 637-649. doi: 10.1007/s00253-011-3773-6.

Huang, X., Ma, T., Tian, J., Shen, L., Zuo, H., Hu, C., Liao, G. (2017). wblA, a pleiotropic regulatory gene modulating morphogenesis and daptomycin production in Streptomyces roseosporus. *Journal of Applied Microbiology*, **123**(3). doi: 10.1111/jam.13512.

Lee, S.K., Kim, H.R., Jin, Y.Y., Yang, S.H., Suh, J.W. (2016) Improvement of daptomycin production via increased resistance to decanoic acid in Streptomyces roseosporus. *J Biosci Bioeng*, **122**(4), 427-33. doi: 10.1016/j.jbiosc.2016.03.026.

Liao, G., Wang, L., Liu, Q., Guan, F., Huang, Y., Hu, C. (2013). Manipulation of kynurenine pathway for enhanced daptomycin production in Streptomyces roseosporus. *Biotechnol Prog*, **29**(4), 847-52. doi: 10.1002/btpr.1740.

Lu, W., Fan, J., Wen, J., Caiyin, X.Q. (2011). Kinetic Analysis and Modeling of Daptomycin Batch Fermentation by Streptomyces roseosporus. *Applied Biochemistry&Biotechnology*. doi: 10.1007/s12010-010-9053-6.

Luo, S., Chen, X.A., Mao, X.M., Li, Y.Q. 2018. Transposon-based identification of a negative regulator for the antibiotic hyper-production in Streptomyces. *Appl Microbiol Biotechnol*, **102**(15), 6581-6592. doi: 10.1007/s00253-018-9103-5.

Mao, X.M., Luo, S., Li, Y.Q. (2017). Negative regulation of daptomycin production by DepR2, an ArsR-family transcriptional factor. *Journal of Industrial Microbiology and Biotechnology*, **44**(12), 1-6. doi: 10.1007/s10295-017-1983-3.

Ng, I.S., Ye, C., Zhang, Z., Lu, Y., Jing, K. (2014). Daptomycin antibiotic production processes in fed-batch fermentation by Streptomyces roseosporus NRRL11379 with precursor effect and medium optimization. *Bioprocess Biosyst Eng*, **37**(3), 415-423. doi: 10.1007/s00449-013-1007-2.

Penn, J., Xiang, L., Whiting, A., Latif, M., Gibson, T., Silva, C.J., et al. (2006). Heterologous production of daptomycin in Streptomyces lividans. *Journal of Industrial Microbiology & Biotechnology*, **33**(2), 121-128. doi: 10.1007/s10295-005-0033-8.

Yu, G., Hu, Y., Hui, M., Chen, L., Wang, L., Liu, N., et al. (2014). Genome Shuffling of Streptomyces roseosporus for Improving Daptomycin Production. *Applied Biochemistry&Biotechnology*. doi: 10.1007/s12010-013-0687-z.

Yu, G., Jia, X., Wen, J., Lu, W., Wang, G., Caiyin, Q.,et al. (2011a). Strain Improvement of Streptomyces roseosporus for Daptomycin Production by Rational Screening of He–Ne Laser and NTG Induced Mutants and Kinetic Modeling. *Appl.biochem.biotechnol*, **163**(6), 729-743. doi: 10.1007/s12010-010-9078-x.

Yu, G., Jia, X., Wen, J., Wang, G., Chen, Y. (2011b). Enhancement of daptomycin production in Streptomyces roseosporus LC-51 by manipulation of cofactors concentration in the fermentation culture. *World Journal of Microbiology & Biotechnology*, **27**(8), 1859-1868. doi: 10.1007/s11274-010-0644-8.

Yuan, P.H., Zhou, R.C., Chen, X., Luo, S., Wang, F., Mao, X.M., et al. (2016). DepR1, a TetR Family Transcriptional Regulator, Positively Regulates Daptomycin Production in an Industrial Producer, Streptomyces roseosporus SW0702. *Appl Environ Microbiol*, **82**(6), 1898-1905. doi: 10.1128/AEM.03002-15.
