# Supplementary material for: Improving the Yield and Quality of Daptomycin in Streptomyces roseosporus by Multilevel Metabolic Engineering
Source: Front Microbiol. 2022 Apr 18;13:872397. doi: 10.3389/fmicb.2022.872397 (PMC9058172; doi:10.3389/fmicb.2022.872397)
Supplement: Supplementary file 6 [file Data_Sheet_2.docx]

**Supplementary Figure Legends**

**Figure S1. Schematic diagram of the daptomycin biosynthesis pathway.**

DptE, an acyl AMP ligase; DptF, an acyl carrier protein ; DptA, DptBC and DptD are Non-ribosomal peptide synthetases (NRPSs) .The dashed line surrounds the amino acid or decanoyl incorporated by enzymes above.

**Figure S2.** **Identification of recombinant plasmids 701DIAA and 702DIAAS .**

(a) PCR with primer pairs L1(primers 38/39) and R1(primers 40/41) to determine the insertion of attP-integrase-AMP cassette into pBeloBac*::*dpt* for generating 701DIAA recombinant plasmid; (b)PCR with primer pairs L2(primers 42/43) and R2(primers 44/45) to determine the insertion of homologous recombination fragment containing spectinomycin gene into701DIAA for generating recombinant plasmid 702DIAAS with plasmid 701DIAA as a control ; PCR with primer pairs L1(primers 38/39) and R1(primers 40/41) to confirm the existence of attP-integrase-AMP cassette in plasmid 702DIAAS again with plasmid 701DIAA as a control.

**Figure S3. Identification of gene deletion and insertion in recombinant strains by**

**PCR.** (a) PCR with primers 46/47 to determine the deletion of *orf3244*; (b) PCR with primers 48/49 to determine the deletion of *orf3242*; (c) PCR with primers 50/51 to determine the integration of pSOK804-ermEp*-*dptJ-orf3245-orf3343* in attB_VWB_ locus; (d) PCR with primers 52/53 to determine the deletion of *phaR*; (e) PCR with primers 54/55 to determine the deletion of *arpA*; (f) PCR with primers 56/57 to determine the deletion of *orf3265-orf3266*; (g) PCR with primer pairs 58/59(for upstream identification of daptomycin biosynthesis gene cluster introduced from outside) and 60/61(for downstream identification of daptomycin biosynthesis gene cluster introduced from outside) to determine the integration of *702DIAAS* in attB_φ31_ locus; (j) PCR with primers 62/63 to determine the integration of *vgb2* in attB_VWB_ locus; M: DNA Marker; + : Positive strain; -: Unknockout strain(a, b, d, e, f); Uninsertion strain (c, g, h).

**Figure S4.** **Concentration of kynurenine (Kyn) and tryptophan (Trp) in the cytoplasm.** The mycelia cultivated in YEME medium were harvested each 24h. The [content](file:///C:\%E7%A8%8B%E5%BA%8F\%E6%9C%89%E9%81%93%E8%AF%8D%E5%85%B8\Dict\7.2.0.0511\resultui\dict\?keyword=content) of kynurenine and tryptophan was measured by HPLC and the [content](file:///C:\%E7%A8%8B%E5%BA%8F\%E6%9C%89%E9%81%93%E8%AF%8D%E5%85%B8\Dict\7.2.0.0511\resultui\dict\?keyword=content) of total protein was measured by Bradford method. The concentration was determined with dividing [content](file:///C:\%E7%A8%8B%E5%BA%8F\%E6%9C%89%E9%81%93%E8%AF%8D%E5%85%B8\Dict\7.2.0.0511\resultui\dict\?keyword=content) of kynurenine and tryptophan by [content](file:///C:\%E7%A8%8B%E5%BA%8F\%E6%9C%89%E9%81%93%E8%AF%8D%E5%85%B8\Dict\7.2.0.0511\resultui\dict\?keyword=content) of total protein.

**Figure S5. The HEATMAP for EC2.6.1.7 according to the local blast results.** NP_012475, a [kynurenine--oxoglutarate transaminase](https://www.ncbi.nlm.nih.gov/protein/NP_012475.3/) from *Saccharomyces cerevisiae* S288C; AM113472-TioG, a [kynurenine aminotransferase](https://www.ncbi.nlm.nih.gov/protein/CAJ34363.1/) from *Micromonospora sp.* ML1; AAF10201, an [aspartate aminotransferase](https://www.ncbi.nlm.nih.gov/protein/AAF10201.1/) from *Deinococcus radiodurans* R1; Orf3256, Orf2688, Orf2371, Orf5777, Orf3248, Orf1390, Orf2174, Orf5673 and Orf3508 are proteins encoded by the corresponding genes in the genome of *Streptomyces roseousporus* and got relatively high scores during the local blast results.

**Figure S6. The growth curve of strain L2797 and L2797-VHb under the conditions of simulated low dissolved oxygen in the shake flask.** The mycelia were cultivated in 75ml YEME medium in a 250ml Erlenmeyer flask. The shaker speed was set to a low speed that is 100rpm. 1mL of culture was harvested each 24h. The mycelia were washed twice by 0.9% sodium chloride aqueous solution and then used to analysis of the mycelia dry weight.
