# Supplementary material for: Improving the Yield and Quality of Daptomycin in Streptomyces roseosporus by Multilevel Metabolic Engineering
Source: Front Microbiol. 2022 Apr 18;13:872397. doi: 10.3389/fmicb.2022.872397 (PMC9058172; doi:10.3389/fmicb.2022.872397)
Supplement: Supplementary file 7 [file Data_Sheet_3.docx]

**Shake-flask and scale-up fermentation Process**

The shake-flask fermentation of *S. roseosporus* strains was partially similar to the previously described (Wang et al., 2014). In brief, Spores of *S. roseosporus* L2790 and its derivatives were prepared from R5 medium and then stored in liquid containing 20% glycerol at -80℃. When shake-flask fermenting, 100 μL of the seed preservation solution stored at -80℃ was used to inoculate in 35 mL TSB. The cultures were grown at 30℃ on a shaker through an arc of 26 cm at 250 rpm for 48h. 0.5 mL of such a culture was inoculated into 35 mL of second-stage TSB medium and then it was cultured in conditions described supra for 24 h. 1 mL of the second-stage culture was used to inoculate 35 mL of YEME medium and then fermented under the conditions described above for 7 days. All media above were placed in a 250 mL Erlenmeyer flask. From 72 h to the end of fermentation, the precursor solution was added to the culture in the proportion of one-thousandth (V/V) every 12 h (Wang et al., 2014).

The fermentation in fermenter of *S. roseosporus* strains was also partially similar to the previously described (Yuan et al., 2016). In brief, 100 μL of the seed preservation solution stored at -80℃ was used to inoculate 35 mL TSB-MD medium for 48h. 1 mL of the culture was inoculated into 35 mL of second-stage TSB-MD medium for culturing 24h. Conditions of culturing were same as described above. 6 mL of the culture was then inoculated into 150 mL of the secondary seed medium in a 1 L Erlenmeyer flask and a total of 3 flasks were inoculated, with a total volume of 450 mL. The flasks were shaken at 250 rpm through an arc 26 millimeters in diameter and at 30°C for about 36h. Then 450 mL of the secondary seed culture was inoculated into 9 L of fermentation medium in a 15 L fermenter. Mycelia were cultured for further 11 days under the following conditions, 30℃, 10 L/min air flow, 0.03 MPa fermenter pressure, during the first 12h, 200 rpm agitation, after that, dissolved oxygen (DO) was controlled through cascade control associated with stirring speed until the end of fermentation and the dissolved oxygen threshold is set to 30%. In this process, feeding medium was added after 96h of culturing at a rate of 0.1-0.15 mL/L·h during 96-120h and 0.2 mL/L ·h from 120h to the end. All fermenters were manufactured by Shanghai Guoqiang Biological Engineering Equipment Co., Ltd.

**References**

WANG, F., REN, N. N., LUO, S., CHEN, X. X., MAO, X. M. & LI, Y. Q. (2014). DptR2, a DeoR-type auto-regulator, is required for daptomycin production in *Streptomyces roseosporus*. *Gene.* 544**,** 208-215. doi: 10.1016/j.gene.2014.04.044.

Yuan, P.H., Zhou, R.C., Chen, X., Luo, S., Wang, F., Mao, X.M., et al. (2016). DepR1, a TetR Family Transcriptional Regulator, Positively Regulates Daptomycin Production in an Industrial Producer, Streptomyces roseosporus SW0702. *Appl Environ Microbiol*, **82**(6), 1898-1905. doi: 10.1128/AEM.03002-15.
